# Advantages of Active Association Membership1Read before the Missouri Valley Veterinary Medical Association, October 5, 1898.

**Published:** 1899-02

**Authors:** S. Stewart

**Affiliations:** Kansas City, Kansas


### ADVANTAGES OF ACTIVE MEMBERSHIP IN A VETERINARY ASSOCIATION.[1]

By Dr. S. Stewart,
KANSAS CITY, KANSAS.

A veterinarian naturally communicates his thoughts concerning his professional labors to some one or more persons with whom he mingles, detailing his successes, discoveries, and difficulties; also accepts and applies suggestions from such persons. If a layman rather than a veterinarian be such confidant, it is doubtful that he

[1] Read before the Missouri Valley Veterinary Medical Association, October 5, 1898.

will be greatly benefited by such communications, not having the needful professional training to comprehend it, nor are his suggestions likely to have much professional value. Quite different must be the results if this converse be with veterinarians. Association supplies the opportunities for giving and receiving of that knowledge which makes one strong in his chosen profession.

It is rarely, indeed, that a man is found who rightly estimates his own talents. If he be a pessimist he will fear that his capabilities are not sufficient to render good service, that he cannot perform a surgical operation as successfully and as deftly as another surgeon with whom he competes; that he cannot state his ideas as clearly and as intelligently as his colleagues in another city. On the other hand, the optimist is wont to conclude that he has accomplished wondrous successes where others would probably have failed, and what he does not know concerning the medical or surgical art is not to be learned from his neighbor. By association for the discussion of professional topics the veterinarian finds opportunity to discover each his own abilities, as well as that of others. Each finds that his talents are neither so mean nor so great that he cannot both teach the most erudite and learn from those whose opportunities have not been all they have desired.

It is very helpful to most men to learn that they do not alone possess the sum total of veterinary wisdom in their community, while the over-modest and timid are materially strengthened upon finding that their knowledge or method is meritorious and compares favorably with that of others. While each discovers in part his own shortcomings, he learns that his associate veterinarians are very human, just like himself, having much to learn yet something to contribute to the mutual advancement of all.

That our beloved profession is in an active, growing state must be patent to every veterinarian, and if he would keep apace he must utilize every facility for acquiring information, not the least valuable of which (I believe the most valuable) is the veterinary association. None of us has time to observe everything, test every remedy, study out and determine every disease or condition. Each may make some observation of professional value, determine the efficiency of some special medicament or plan of treatment, discover the cause and nature of some disease, and by communicating what he has learned to his associates he contributes to the knowledge of others to their mutual advantage. If his contribution be supplied to the veterinary journals for publication the profession at large is benefited.

If it happens that one's observations are incomplete or one's methods of investigation faulty, and wrong conclusions deduced, he may be set aright through friendly criticism of his methods or deductions, by those associated with him and who will extend personal interest and sympathy in his efforts to arrive at the more complete understanding of the subject of his study. Let me illustrate this point: We may have read in the journals or heard some veterinarian say that it now seems probable that the very common yet intractable disease of cattle generally known as milk-fever, or parturient apoplexy, is dependent upon a micro-organismal infection of the udder, and a fairly successful treatment consists of the local application of a solution of iodide of potassium to the udder by injection through the teats. I may apply the remedy in the next ten cases and have a death-rate of 60 per cent. Naturally my conclusion will be that the treatment is no more successful than that employed in former cases, and my opinion will be quite strong that the asserted cause of the disease is erroneous. Two or more of you may have tried the same remedy, and have been very successful with it, saving all your cases. One will present the subject in the form of a paper before the Association. By the discussion of this paper I will be able to discover that my method was faulty, and hence my failures and my erroneous opinion. He who attends the associations and who takes an active part in the proceedings is the one who grows with his profession, and helps place his profession on a higher plane; who lives up to his opportunities and best serves his community; who enlarges his capacity for relieving the ills of animals entrusted to his care, and correspondingly increases his revenues.

The veterinarian who says to himself I will open an office in this place; I will post my sign and let the people know that I am prepared to look after their sick animals better than others are doing; I will not rob them of unearned fees like others are reported to have done; I will not resort to such unscrupulous methods it is said others do; I will attend strictly to my own affairs, the getting of clients and dollars, and will have nothing to do with other veterinarians, as they doubtless are quacks. This veterinarian will probably find that business does not hastily seek out the self-announced honest practitioner; that competitors continue to have clients, and apparently do a thriving business, and if he will but study himself he will find a large development of sordid selfishness with a tendency to contribute to the malignant reports concerning his competitors, and that he is even resorting to methods which he at first condemned. If this veterinarian but takes the opposite course, and courts the acquaintances of neighboring veterinarians and associates with them for the discussion of professional topics and mutual interests, he will find they are not such illiterate and professional monsters that irresponsible and misguided persons have pictured them to be. He will doubtless find they are applying their professional knowledge with the hope of best results, and are endeavoring to obtain an honest living as that term is usually understood. If they have come short of maintaining the highest ideals of professional practice it is largely due to lack of conviction and, perhaps, perception of the best ideals and best practical methods of ethical procedure, due to lack of a common understanding among the several veterinarians in a community as to what rules should guide them in their relations to each other and to the public. It is only by association that wholesome professional practice is established and maintained.

The well-qualified veterinarians most keenly appreciate the losses and disappointments suffered by the public through confidence placed in the claims of untrained, uneducated, designing persons who advertise to be skilled veterinarians and are entrusted with the medical care of the favorite horse, the much-needed cow, or the highly prized dog. They exact large fees for valueless service and lead the uninformed to underestimate the merits and worth of the competent practitioner. Laws restraining the incompetent practitioner and the fraudulent pretender should be enacted for the protection alike of the public and the profession. Such legislation cannot be secured until the subject is agitated by those most interested, and suitable bills framed and their passage secured through the intelligent, well-directed efforts of veterinarians who have studied the problem and best comprehend the provisions of law which will accomplish the end sought. It is only by association that veterinarians can reach a mutual understanding and secure concerted action for the procurement of legislation regulating veterinary practice or promote the cause of veterinary sanitary regulations in municipalities and states.

It is an old truism handed down from past ages that it is not meet that man should be alone. While this truism expresses a factor inherent in man as a social being, it is also true relative to his vocation, especially if it be a profession. He is indebted to the past for that accumulated stock of knowledge wrought out of experience and patient observation, to which he had access and upon which he has so largely and freely drawn, and in return for which he is under moral obligation to add what he can to this general fund of knowledge for the use of coming generations. Organizations of individuals for the advancement of any particular profession is one of the best means for accomplishing definite progress, and has the advantage of imparting stimulation to latent powers and talents which might otherwise remain dormant and sterile.

The veterinary profession is not different from other professions in relation to this general principle. The veterinarian can work to better purpose and to nobler end if he be encouraged and stimulated by the coöperation and sympathy of his fellows. Association with others having the same vocation develops and strengthens the bonds of brotherhood; tends toward a larger conception of professional duty and amenity; gives incentive to study and scientific reflection; encourages honesty of purpose, better business methods, manliness and self-respect; affords a higher quality of companionship, and that attrition, that intellectual friction, which leads to better thinking and a more perfect application of veterinary science, and provides an intelligent, forceful body to work for higher and better sanitary and veterinary practice regulations.

